# A Comparative Study of the Use of Mesoporous Carbon and Mesoporous Silica as Drug Carriers for Oral Delivery of the Water-Insoluble Drug Carvedilol

**DOI:** 10.3390/molecules24091770

**Published:** 2019-05-07

**Authors:** Cuiyan Han, Haitao Huang, Yan Dong, Xiaoyu Sui, Baiyu Jian, Wenquan Zhu

**Affiliations:** College of Pharmacy, Qiqihar Medical University, Qiqihar 161006, China; hcycjy2013@163.com (C.H.); hhtcq@sohu.com (H.H.); dongyan00@hotmail.com (Y.D.); suixiaoyu@outlook.com (X.S.); abcaaa1@126.com (B.J.)

**Keywords:** mesoporous silica, mesoporous carbon, carvedilol, in vitro dissolution, water–insoluble drug

## Abstract

Mesoporous carriers have been extensively applied to improve the dissolution velocity and bioavailability of insoluble drugs. The goal of this work was to compare the drug-loading efficiency (LE) and drug-dissolution properties of mesoporous silica nanoparticles (MSN) and mesoporous carbon nanoparticles (MCN) as drug vectors oral delivery of water-insoluble drugs. For this purpose, MSN and MCN with similar particle size, surface area, and mesoporous diameter were prepared to precisely evaluate the effects of different textures on the drug-loading and dissolution behavior of insoluble drugs. Carvedilol (CAR), a Bio-pharmaceutic Classification System (BCS) class II drug, was loaded in the MSN and MCN by the solvent adsorption method and solvent evaporation method with different carrier–drug ratios. The carboxylated MCN (MCN–COOH) had a higher LE for CAR than MSN for both the two loading methods due to the strong adsorption effect and π–π stacking force with CAR. In vitro drug dissolution study showed that both MSN and MCN-COOH could improve the dissolution rate of CAR compared with the micronized CAR. In comparison to MSN, MCN-COOH displayed a slightly slower dissolution profile, which may be ascribed to the strong interaction between MCN-COOH and CAR. Observation of cell cytotoxicity and gastrointestinal mucosa irritation demonstrated the good biocompatibility of both MSN and MCN–COOH. The present study encourages further research of different carriers to determine their potential application in oral administration.

## 1. Introduction

With the development of high–throughput screening technology, many new chemical drugs have been developed [1,2,3]. It has been reported that more than 40% of new active substances cannot be used clinically because of their poor water solubility, resulting in low oral bioavailability and problems with oral absorption [4,5,6,7,8]. Therefore, in order to transform more insoluble candidate compounds into new therapeutic drugs and maximize their efficacy, enhancing the solubility and bioavailability of these drugs is an important scientific problem that urgently needs to be solved.

Oral drug delivery is a preferred administration route for most drugs due to its advantages such as better compliance, lower cost, and fewer safety problems compared with intravenous delivery [1,2,3]. It is well known that more than one-third of candidate compounds are insoluble in aqueous solution, which might lead to poor oral absorption in the gastrointestinal (GI) tract [4]. The water-insoluble drugs which belong to the Bio-pharmaceutic Classification System (BCS) class II have the characteristics of high permeability and low solubility. The dissolution of water insoluble drugs in the GI tract is a prerequisite for transmembrane transport after oral administration. Thus, the dissolution step throughout the GI tract is the rate-limiting process which decides the oral absorption of poorly water-soluble drugs [5]. Hence, increasing the dissolution velocity and solubility are essential to improving the oral adsorption of water insoluble drugs. 

Solid dispersion technology is a promising strategy to improve the oral bioavailability of insoluble drugs [6,7]. Inorganic porous materials including Aerosil, Syslia and Parteck have been extensively applied as drug carriers for solid dispersions [8]. Over the last few decades, inorganic mesoporous materials have attracted extensive attention because they have several fascinating features including, high strength, uniform pore size, large surface area, high bio-compatibility and chemical inertness [9,10]. The loading of water-insoluble drugs in inorganic mesoporous materials could markedly decrease the particle size of the drugs, increase the surface area and porosity, and improve wettability. In addition, insoluble drugs loaded in a mesoporous carrier will possess faster dissolution velocity due to the non-crystalline state, which could remarkably increase oral bioavailability [11]. Among the inorganic mesoporous materials, mesoporous silica nanoparticles (MSN) and mesoporous carbon nanoparticles (MCN) have received extensive attention owing to their tunable pore size and high pore volume in addition to the advantages mentioned above [12,13,14,15]. They could offer large storage capacity for drug molecules. Many studies in the literature have reported MSN or MCN alone used as a drug carrier for water-insoluble drugs [16,17,18]; however, a comparative study of MSN and MCN used as drug carriers to load the same water-insoluble drug has not yet been reported. 

**Figure 1:**
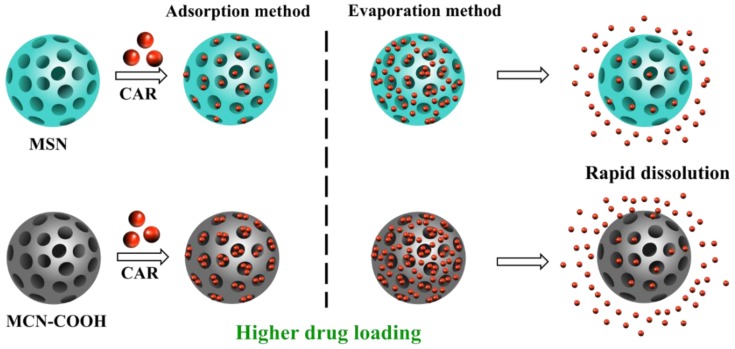
Schematic illustration of mesoporous silica nanoparticles (MSN) and mesoporous carbon nanoparticles (MCN)–COOH as the carriers for water–insoluble drug carvedilol by solvent adsorption method and solvent evaporation method.

The particle size, surface area, mesoporous size, and pore structure of the carriers might have an important effect on the drug-loading efficiency (LE) and dissolution rate. In addition, the different drug-loading methods also play a vital part in regulating the loading and dissolution properties of insoluble drugs. Therefore, in order to study precisely the effects of different carriers on the drug-loading and dissolution behavior of insoluble drugs, the prepared MSN and MCN carriers should have the similar structure parameter and particle size. For this purpose, MSN and MCN with similar parameters were carefully prepared as shown in Figure 1. Carvedilol (CAR), a BCS class II drug, widely used for the treatment of cardiovascular diseases, was loaded into the MSN and MCN. Different drug loading methods and different carrier-drug ratios were studied to evaluate the dissolution and drug loading properties of CAR-loaded MSN and MCN. Various characterization methods were chosen to characterize the blank mesoporous carriers and CAR-loaded mesoporous carrier formulations with the aim to investigate the effects of MSN or MCN carriers on the LE and dissolution rate and the possible mechanism for drug-dissolution improvement.

## 2. Results and Discussion 

### 2.1. Preparation and Characterization of Mesoporous Silica Nanoparticles (MSN) and Mesoporous Carbon Nanoparticles (MCN)–COOH

**Figure 2:**
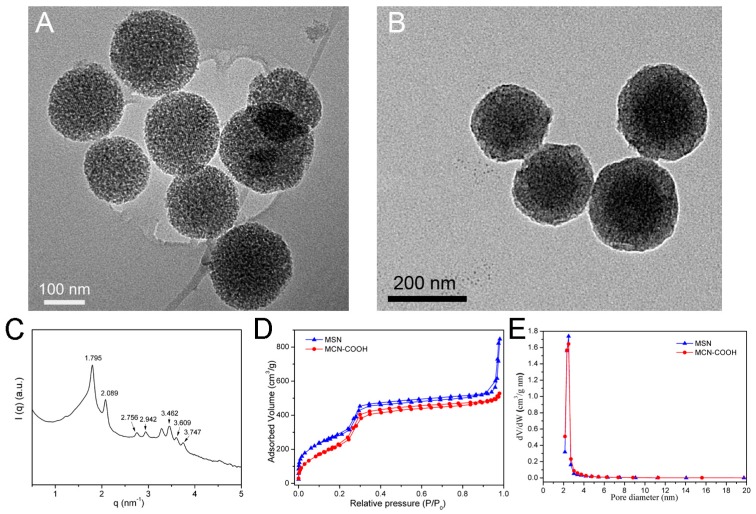
Transmission electron microscope (TEM) image of (**A**) MSN and (**B**) MCN-COOH; (**C**) small angle X-ray scattering (SAXS) pattern of MSN; (**D**) Nitrogen adsorption/desorption isotherms and (**E**) pore size distributions of MSN and MCN-COOH.

The particle size, surface area and mesoporous size of the carriers have an important effect on the drug loading and dissolution behavior. Hence, MCN and MSN with similar structure parameters and particle size were carefully prepared. The MCM–48-type MSN were prepared using cetyltrimethylammonium bromide (CTAB) as a template and F127 as a dispersion stabilizer. The MCN were prepared using resorcinol as the carbon source and hexadecyl trimethyl ammonium chloride (CTAC) and tetraethoxysilane (TEOS) as the structure–directing agent. After the samples were calcined for carbonization, the C–Si compounds were immersed in hydrofluoric acid (HF) to remove silica in order to obtain the MCN. To obtain a hydrophilic surface, the MCN were oxidized using concentrated H_2_SO_4_ and ammonium persulfate. As shown in Figure 2A, the monodispersed and spherical MSN with an average diameter of 200 nm were prepared, and the highly ordered mesoporous channel could be clearly observed. The average diameter of MCN–COOH was 200 nm with uniform particle size and highly ordered mesopores shown in the transmission electron microscope (TEM) image (Figure 2B). Additionally, small angle X-ray scattering (SAXS) of MSN was carried out to confirm the pore order. SAXS pattern (Figure 2C) of MCM48-type MSN showed the seven characteristic peaks, proving a three-dimensional mesopore channels of Ia 3d symmetry [19]. Hence, MSN had a similar diameter and appearance to MCN–COOH.

The surface area and the pore distributions of mesoporous carriers play a crucial role in regulating the drug loading and dissolution properties. The isotherms and pore size distribution profiles were evaluated by N_2_ adsorption and desorption analysis (Figure 2C,D). The parameters of the Brunauer–Emmett–Teller (BET) surface area (S_BET_), pore volume (V_P_), and pore size distribution (P_d_) are shown in Table 1. The S_BET_ and V_P_ of MSN were 1042 m^2^/g and 0.854 cm^3^/g, respectively, and the P_d_ was 2.6 nm. The S_BET_ and V_P_ of MCN–COOH were 923 m^2^/g and 0.857 cm^3^/g, and the P_d_ was also 2.6 nm. These results indicated that the MSN had similar S_BET_, V_P_, and P_d_ values when compared with MCN–COOH, which was very important to comparing the drug loading and dissolution behavior for MSN and MCN–COOH. 

**Figure 3:**
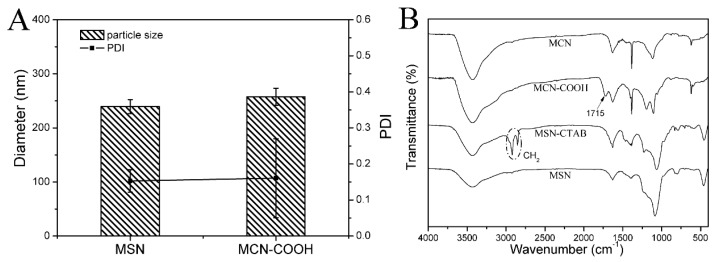
(**A**) The corresponding zeta potentials and particle sizes of MCN–COOH and MSN; (**B**) the Fourier transform–infrared (FT–IR) spectra of MCN, MCN–COOH and MSN.

The particle sizes and corresponding zeta potentials of MSN and MCN–COOH were also measured as shown in Figure 3A. The particle size of MSN was 240 nm with a polydispersity index (PDI) of 0.129, which was larger than that of the TEM result. The size of the nanoparticles observed by TEM was in the dried condition without the hydration layer, while, the hydrodynamic diameter of the sample characterized by dynamic light scattering (DLS) was in water condition. MCN–COOH had a similar particle size to MSN of 258 nm. Furthermore, the corresponding zeta potentials of MCN–COOH and MSN were –21.5 mV and –33.8 mV and were attributed to the silanol groups in MSN and the carboxyl groups in MCN–COOH. A comparison of the Fourier transform–infrared (FT–IR) spectra of MCN, MCN–COOH, MSN–CTAB, and MSN are shown in Figure 3B. Compared with MCN, the appearance of a characteristic C=O absorption band at 1715 cm^–1^ for MCN–COOH indicated that carboxyl groups had been introduced in MCN–COOH. The peaks around 2852 and 2922 cm^−1^ were owing to the vibration of –CH_2_ belonging to the CTAB surfactant, and the –CH_2_ vibration peaks disappeared after the removal of the surfactant by calcination. 

### 2.2. Drug Loading by Different Loading Methods

The melt method, solvent adsorption method and solvent evaporation method are the most common methods of drug loading for porous carriers. However, some works have reported that the melt method is not very suitable for loading insoluble drugs on the mesoporous carriers; this is because drugs with high viscosity in a molten state did not flow easily into the mesopores of carriers within a short time period [20]. Most of these drugs formed a drug molecular layer and were coated on the surface of mesoporous carriers [20]. 

#### 2.2.1. Drug Loading by the Solvent Adsorption Method

For the solvent adsorption method, attaining the adsorption equilibrium is the main procedure for drug loading. The drug molecules were given enough time to enter through the mesoporous network of the carriers during the loading process. Then, the unbound drug was easily removed by the filtration or centrifugation process, and a uniform distribution of the drug was easily obtained. The advantage of the solvent adsorption method is that the adsorption process is carried out in a high concentration of the drug solution, and there is no need to screen the ratio of carrier to drug. The LE of MSN/CAR was disappointingly low—just 7.9%. The drug-adsorption process would finish after the adsorption equilibrium was attained; hence, the solvent adsorption method had a relatively low LE. However, the LE of MCN–COOH/CAR was 16.7%, which is much higher than that of MSN/CAR. The reason for this is that MCN–COOH had a stronger adsorption capacity compared with MSN and supermolecular π–π stacking for high drug loading. 

**Figure 4:**
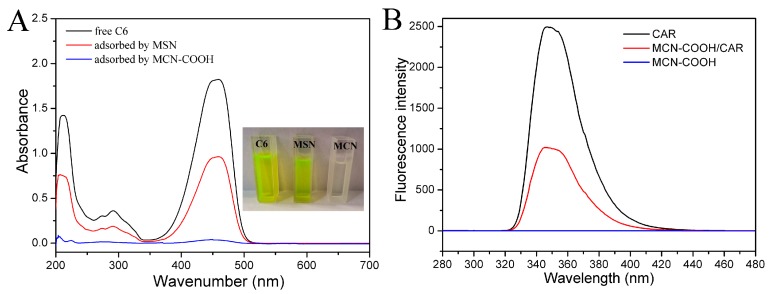
(**A**) Photograph of the supernatant after MCN–COOH and MSN adsorption and the original C6 solution, (**B**) fluorescence emission spectra of Carvedilol (CAR), MCN–COOH/CAR and MCN–COOH solution with an excitation wavelength at 254 nm.

In order to confirm that MCN–COOH had a stronger adsorption capacity, a hydrophobic dye, Coumarin 6, was used to simulate the drug adsorption process. As displayed in Figure 4A, a color change of the supernatant after MCN–COOH adsorption was more obviously observed than after that of MSN. The supernatant was nearly colorless after being adsorbed by MCN–COOH, while it remained bright yellow fluorescence after being adsorbed by MSN under the same conditions. This study showed that MCN–COOH had stronger adsorption ability than MSN because of the strong adsorption capacity of carbonaceous material. It has been reported that the MCN have the supermolecular π–π stacking interaction with aromatic drugs [21]. The interaction between CAR and the MCN–COOH carrier was evaluated using a microplate reader (BIO–RAD680, iMark, Hercules, CA, USA). The concentration of CAR for the CAR and MCN–COOH/CAR samples was at 10 μg/mL. CAR, MCN–COOH/CAR, and MCN–COOH in deionized water containing 2% methanol were measured by fluorescence spectrum at an excitation wavelength at 254 nm. Compared with MCN–COOH, free CAR exhibited an intense emission peak at 350 nm as shown in Figure 4B, while the fluorescence intensity of MCN–COOH/CAR showed obvious quenching at the emission band with the same excitation wavelength. The fluorescence quenching phenomenon was due to the strong π–π stacking interaction between MCN–COOH and CAR [22].

#### 2.2.2. Drug Loading by the Solvent Evaporation Method

Unlike the solvent adsorption method, the drug loading process for the solvent evaporation method is continuous with the evaporation of the solvent, and nearly all of the drug was entrapped in the mesopores and adsorbed on the surface of carriers. Therefore, the solvent evaporation method had a higher LE compared with the adsorption method. The appropriate ratio of carrier to drug was also very important for the solvent evaporation method. When the ratio was too high, the drug LE was relatively low and it was hard to meet the clinically required dose. When the ratio was too low, the drug could not be completely loaded in the mesopores and on the surface of the carriers, and some of the drug could crystallize on the surface of the carriers. Therefore, different ratios of carrier to drug were chosen to optimize the drug loading condition, as shown in Table 2. With the increased mass of the carrier, the LE of CAR was also increased for both the MSN and MCN–COOH. The LE of CAR was slightly lower than the theoretical drug loading, which was due to a portion of the drug adhering to the container. Hence, to obtain a higher LE to meet the clinically required dose, the solvent evaporation method was chosen in the following test.

The theoretical *LE*(%) was calculated according to the following formula.

$$LE(\%)=\frac{m_{CAR}}{m_{CAR}+m_{1}}$$

where *m_CAR_* is the mass of CAR and *m*_1_ is the mass of the carrier.

### 2.3. Solid State Characterization by X-ray Diffraction (XRD) and Differential Scanning Calorimetry (DSC)

The crystalline properties of the raw drug and different carrier preparations were verified by XRD and DSC. The carrier-to-drug ratio is especially important for the solvent evaporation method. When the proportion of the carrier is too low, CAR cannot be completely loaded in the mesopores of the carrier, and some of the drug precipitates crystals on the surface of the carrier. On the other hand, when the proportion of the carrier is too large, although the drug can be completely loaded into the mesoporous channel of the carriers, the drug-loading amount is too low, which is disadvantageous for efficient loading of the drug. Therefore, different mass ratios of carriers to CAR were chosen in the present work. 

**Figure 5:**
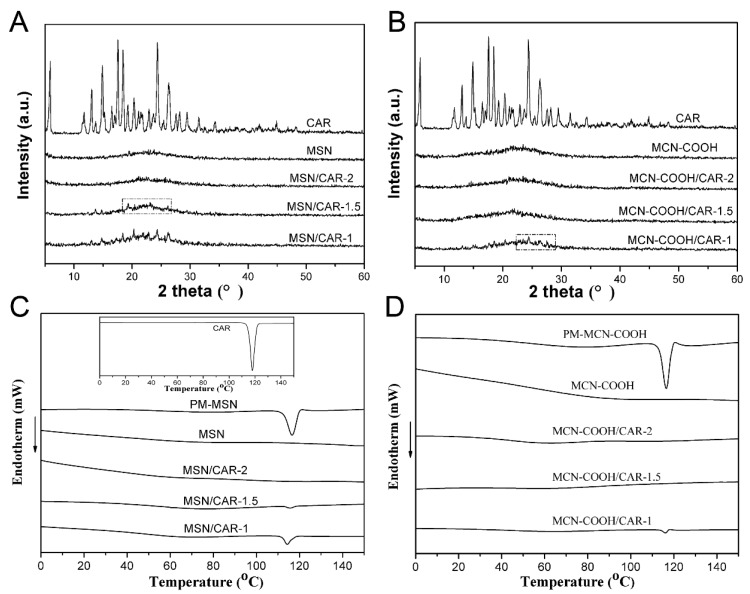
X-ray diffraction (XRD) patterns of (**A**) CAR, MSN, MSN/CAR–1, MSN/CAR–1.5 and MSN/CAR–2; (**B**) CAR, MCN–COOH, MCN–COOH/CAR–1, MCN–COOH/CAR–1.5 and MCN–COOH/CAR–2; DSC curves of (**C**) CAR, physical mixture (PM–MSN), MSN, MSN/CAR–1, MSN/CAR–1.5 and MSN/CAR–2; (**D**) CAR, physical mixture (PM–MCN–COOH), MCN–COOH, MCN–COOH/CAR–1, MCN–COOH/CAR–1.5 and MCN–COOH/CAR–2.

As displayed in Figure 5A, the diffraction pattern of raw CAR exhibited intense and typical diffraction peaks at 2θ = 5.9°, 14.9°, 17.6°, 18.5°, and 24.4°, proving the crystalline structure of CAR. No diffraction peaks of the blank carrier were observed owing to its amorphous nature. However, no crystalline CAR was observed in MSN/CAR–2 (where the mass ratio of MSN to CAR was 2:1). The absence of obvious diffraction peaks indicated that the CAR loaded into the mesopores of MSN was in a non–crystalline state [23]. In contrast, CAR peaks of MSN/CAR–1.5 and MSN/CAR–1 (with mass ratios of MSN to CAR of 1.5:1 and 1:1) were found. This result indicated that crystalline CAR existed due to the weight ratio of MSN to CAR being less than 2:1, and the CAR could not be completely loaded in the mesopores of the MSN carriers. When MCN–COOH was used as the carrier, no crystalline CAR was observed in MCN–COOH/CAR–2 and MCN–COOH/CAR–1.5, proving that the CAR loaded in the mesopores of MCN was in a non–crystalline state. A small crystalline peak was observed for the sample MCN–COOH/CAR–1. 

DSC measurement was further carried out to evaluate the solid state of CAR in different preparations. The melting point peak of raw CAR displayed a sharp peak at 117.1 °C, as shown in Figure 5C. The physical mixture (PM)–MSN showed a similar melting peak at 116.5 °C, attributed to the melting state of CAR. The loaded CAR sample MSN/CAR–1 showed a weaker crystalline peak. Furthermore, for sample MSN/CAR–1.5, a very weak endothermic peak was still observed, indicating the presence of traces of crystalline drug, while no obvious crystalline peak was found in the DSC profile of MSN/CAR–2, indicating that CAR was in a non–crystalline state. Hence, the results of DSC confirmed the results obtained by XRD. The DSC curves of raw CAR, physical mixture (PM–MCN), MCN–COOH, MCN–COOH/CAR–1, MCN–COOH/CAR–1.5 and MCN–COOH/CAR–2 are presented in Figure 5D. The PM–MCN–COOH thermogram also exhibited an intense endothermic peak at 116.5 °C attributed to the fusion point of CAR. The MCN–COOH/CAR–1 sample showed a weak melting peak. Interestingly, no obvious crystalline peak was observed in the DSC curves of MCN–COOH/CAR–2 and MCN–COOH/CAR–1.5, which was also verified by the results obtained by XRD. These results indicated that the optimal mass ratio of MSN to CAR was 1.5:1, while the optimal mass ratio of MCN–COOH to CAR was 2:1. The drug-loading efficiencies of MSN/CAR–2 and MCN–COOH/CAR–1.5 were 27.2% and 34.0%, respectively. 

### 2.4. Drug Dissolution Tests

**Figure 6:**
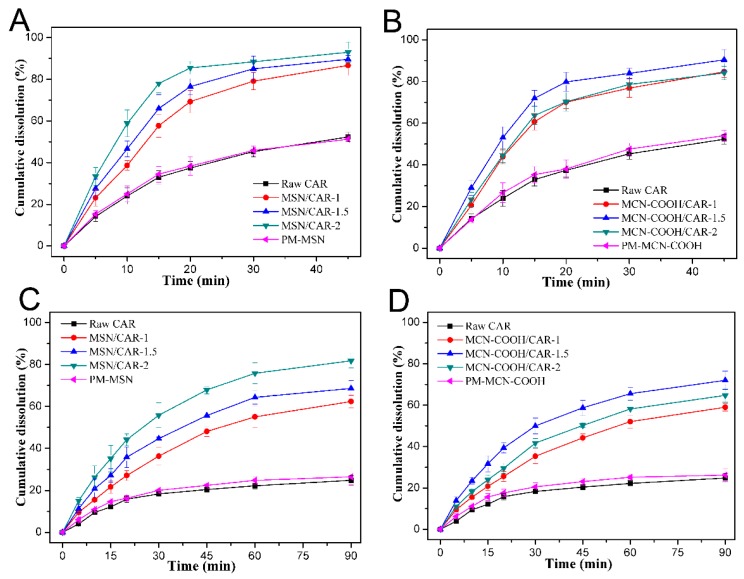
In vitro dissolution profiles of raw CAR, MSN/CAR–1, MCN/CAR–1.5, MCN/CAR–2 and physical mixture (PM)–MSN in (**A**) simulated gastric fluid (SGF) and (**C**) simulated intestinal fluid (SIF); and in vitro dissolution profiles of raw CAR, MCN–COOH/CAR–1, MCN–COOH/CAR–1.5, MCN–COOH/CAR–2 and PM–MCN–COOH in (**B**) SGF and (**D**) SIF.

The CAR dissolution behaviors of mesoporous carrier preparations, including MSN and MCN–COOH with different carrier to drug ratios, were compared to the behaviors of micronized raw CAR drugs in simulated gastric fluid (SGF) and pH 6.8 simulated intestinal fluid (SIF). As shown in Figure 6A, for raw crystalline CAR, only about 50% of CAR was released within 45 min in SGF, indicating the low dissolution rate of the raw drug. Meanwhile, the dissolution behaviors of CAR from the MSN preparations, regardless of the ratios of carrier to drug, were markedly higher than that from raw CAR. In addition, MSN/CAR–2 had the fastest dissolution rate among the three different ratios of preparations; this was ascribed to the fact that all the CAR was in a non–crystalline state in the mesoporous carrier verified by the XRD and DSC results. A similar tendency was also observed in the different MCN–COOH-based preparations (Figure 6B). The cumulative dissolution percentages of CAR from all MCN–COOH preparations were observably higher than those from micronized CAR and PM–MCN–COOH. Surprisingly, MCN–COOH/CAR–1.5 showed the fastest dissolution velocity among the different MCN–COOH preparations. However, the CAR molecules loaded in MCN–COOH/CAR–1.5 and MCN–COOH/CAR–2 were both existed in a non–crystalline state, as proved by the XRD and DSC results, and the MCN–COOH/CAR–1.5 had a higher LE than MCN–COOH/CAR–2 due to the high ratio of MCN–COOH to CAR. That is to say, more of the drug was loaded on the surface of MCN–COOH/CAR–1.5 than on the surface of MCN–COOH/CAR–2. The drug loaded on the surface of MCN–COOH could quickly come into contact with the dissolution medium, thereby showing relatively fast dissolution behavior, while it would take a long time for the drug which was loaded deeply in the mesopores of the carrier to be released into the dissolution medium. Therefore, MCN–COOH/CAR–1.5 had a higher LE and showed a faster dissolution rate than MCN–COOH/CAR–2.

The improved dissolution velocity of CAR by encapsulation in mesoporous carriers would enhance the absorption of CAR in the GI tract, thereby enhancing its oral efficacy [24]. Therefore, dissolution enhancement is vital to improving the oral bioavailability of poorly soluble drugs. Dissolution rate enhancement may be attributed to several reasons: the increased surface area of the loaded drug after being confined in the mesoporous carriers, nanoscale-sized channels of the mesoporous carriers (MSN and MCN) changing the crystalline CAR into a non-crystalline state, and the hydrophilic surface of MSN and MCN–COOH. Furthermore, a marked reduction of particle size into the nanometer range accelerates the dissolution velocity of insoluble drugs [25].

In vitro dissolution tests of micronized CAR and drug-loaded samples were also conducted in SIF, as shown in Figure 6C,D. CAR has pH–dependent solubility due to its weakly basic nature [26]. The dissolution rate of micronized CAR was quite slow in SIF, and the cumulative percentage of dissolved CAR within 90 min only reached about 24%. The physical mixtures of PM–MSN and PM–MCN–COOH both showed similar dissolution properties to crystalline CAR, but the dissolution rates of CAR from all the MCN–COOH preparations markedly improved compared with that from raw CAR. Furthermore, MCN–COOH/CAR–1.5 exhibited the fastest dissolution rate among the different MCN–COOH preparations in SIF. 

**Figure 7:**
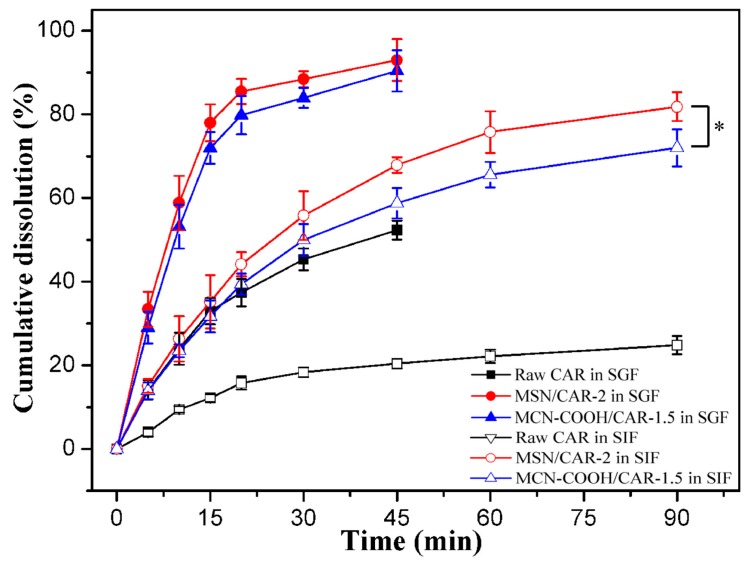
In vitro dissolution profiles of raw CAR, MSN/CAR–2 and MCN–COOH/CAR–1.5 in SGF and SIF. (The * represents significant difference, *p* < 0.05).

The dissolution profiles of raw CAR, MSN/CAR–2, and MCN–COOH/CAR–1.5 in SGF and SIF are shown together in Figure 7. A significant improvement (about 3.5–fold) in the cumulative dissolution rate of MSN/CAR–2 compared with that of micronized CAR was observed in SIF within 90 min, which was more obvious than the increase in SGF. Interestingly, the cumulative dissolution percentage of MCN–COOH/CAR–1.5 was lower than that of MSN/CAR–2 in SIF at 90 min, and the significant differences were found with a *p* value less than 0.05. This may be attributed to the strong π–π stacking interaction between CAR and MCN. Meanwhile, the dissolution rate of MCN–COOH/CAR–1.5 was similar to that of MSN/CAR–2 in SGF, and no significant difference was found. Because CAR is a weakly alkaline drug and has relatively high solubility in SGF, the difference in dissolution between MCN–COOH/CAR–1.5 and MSN/CAR–2 was inconspicuous in SGF. 

### 2.5. In Vitro Cell Cytotoxicity Evaluation

**Figure 8:**
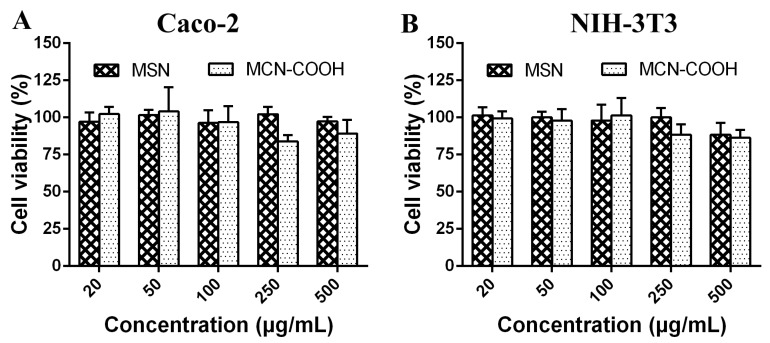
Effect of MSN and MCN–COOH on cell viability of (**A**) caco-2 cells and (**B**) NIH-3T3 cells by MTT assay for 24 h.

The cytotoxicity of MSN and MCN–COOH is an important factor in evaluating their future clinical applications. In vitro cell cytotoxicity assay (MTT) was used to estimate the biocompatibility of MCN–COOH and MSN using Caco-2 cells [27,28] and normal NIH-3T3 cells. The caco-2 cell line is a continuous line of human epithelial colorectal adenocarcinoma cells and is widely used as model cells for oral drug delivery. NIH-3T3 cell is a mouse embryo fibroblast cell line. The result is shown in Figure 8A,B. Negligible cytotoxicity was observed after incubation of caco-2 cells with different concentrations of MSN and MCN–COOH from 20 to 500 μg/mL, and the viability levels of caco-2 cells were 97.2% and 89.1% even at the highest concentration of 500 µg/mL MSN and MCN–COOH, which is a sufficiently high concentration for future applications. Additionally, the cell viability of NIH-3T3 cells incubated with MSN and MCN–COOH for 24 h was above 80% within the tested concentration range. These results indicated that MSN and MCN could be used as safe carriers for oral drug delivery.

### 2.6. Gastrointestinal Mucosa Irritation

**Figure 9:**
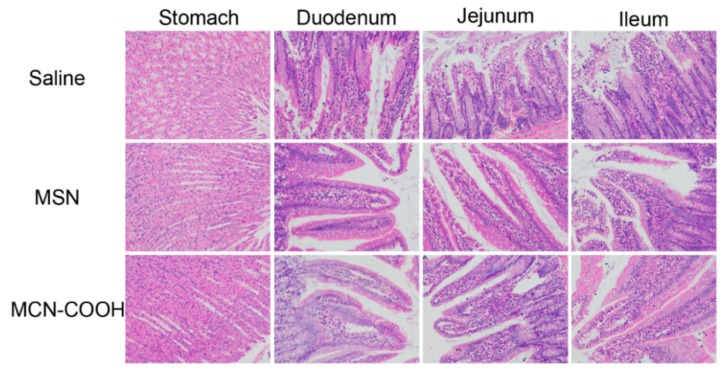
Gastrointestinal mucosa irritation evaluation after oral administration of MSN and MCN–COOH at dose of 100 mg/kg for a week.

A gastrointestinal mucosa irritation test was carried out to evaluate whether the carriers are safe after oral administration. Comparisons were made with a saline group, and no hyperemia or histopathological lesions were observed after oral administration of MSN and MCN–COOH suspension (Figure 9). Furthermore, no unusual behaviors or death of mice were observed during the irritation experiment. Therefore, it was confirmed that the MSN and MCN–COOH showed good tissue compatibility in vivo and could be applied in oral administration.

## 3. Materials and Methods

### 3.1. Chemistry

Tetraethoxysilane (TEOS), cetyltrimethylammonium bromide (CTAB), formaldehyde solution, resorcinol, hexadecyl trimethyl ammonium chloride (CTAC, >99%), ammonium persulfate, and 3-(4,5-Dimethylthiazol-2-yl)-2,5-diphenyl tetrazolium bromide (MTT) were purchased from Aladdin Chemical Inc. (Shanghai, China). Triblock polymer F127 (EO_106_PO_70_EO_106_, M_W_ = 13,400) was purchased from BASF (BASF-YPC Ltd., Hong Kong, China). Cell culture media Dulbecco’s modified Eagle’s medium (DMEM), fetal bovine serum (FBS) and penicillin–streptomycin were supplied by GIBCO, Invitrogen Co. (Carlsbad, NM, USA). Raw CAR was supplied by Shenyang Funing Pharmaceutical Company (Shenyang, China) with a purity >99%. All analytical reagents were not further purified before use.

### 3.2. Preparation of MSN

MCM–48-type MSN were prepared based on the reported method [29]. Briefly, 1 g triblock copolymer F127 and 500 mg CTAB were added in 100 mL water, then 40 mL ethanol and 13 mL concentrated ammonium hydroxide were added. Then, 2 mL TEOS was introduced to the above mixture under stirring, and the reaction was maintained for 1 day. The prepared samples were collected by centrifugation dried in vacuum for 1 day and calcined at 600 °C for 4.5 h to remove the template. The final sample was called MSN.

### 3.3. Preparation of MCN and MCN–COOH

The MCN were prepared according to the published work with some modifications [30]. First, 25 wt % CTAC (1 g) and 0.2 mL concentrated ammonia were added to a mixed solution consisting of 40 mL deionized water and 8 mL ethanol with stirring. After 0.5 h, 0.4 g resorcinol was further dissolved in the above mixture and stirred for 0.5 h. Subsequently, 0.7 mL TEOS and 0.55 mL formaldehyde (37%) were dropwise added to the system, and stirring of the mixture was continued for 1 day. The as–prepared sample was collected by centrifugation, washed with ethanol and distilled water. Then, the solid product was calcined and kept at 750 °C for 2.5 h to induce for carbonization under N_2_ flow. Finally, the MCN were obtained by immersing the Si–C product in 15% hydrofluoric acid (HF) to remove silica. To obtain a hydrophilic surface, carboxyl groups were introduced onto the surface of MCN by the wet oxidation process [31]. About 400 mg MCN was added to a mixed solution consisting of 1.3 mL concentrated sulfuric acid (98 wt%), 2.7 g ammonium persulfate, and 25 mL H_2_O and stirred for 2 h at 65 °C. The final carboxylated MCN were called MCN–COOH.

### 3.4. Drug Loading by Different Methods

#### 3.4.1. Drug Loading by the Solvent Adsorption Method

CAR was chosen as a model drug owing to its low solubility in intestinal fluid and water. Methanol was selected as the solvent in the adsorption equilibrium method due to its lower toxicity and relatively good solubility for CAR. Briefly, 100 mg CAR was dissolved thoroughly in 5 mL methanol, and the same mass of MSN was dispersed in the CAR solution. After the suspension was ultrasonicated for 45 min, the adsorption equilibrium process was gently stirred for more than 8 h to increase the drug loading amount. Subsequently, centrifugation was chosen to get the solid sample. The dried product was called MSN/CAR. When MCN–COOH was used as the drug carrier, the loading process was as the same as that for MSN, and the drug–loaded sample was named MCN–COOH/CAR. 

#### 3.4.2. Drug Loading by the Solvent Evaporation Method

The solvent evaporation method, containing the adsorption equilibrium and following solvent evaporation, was chosen to improve the drug loading amount. Methanol was chosen as the solvent because of its low toxicity and relatively low boiling point. In brief, CAR was loaded in MSN by adding carrier samples into CAR/methanol solution with a concentration of 20 mg/mL. The weight of CAR was fixed at 100 mg, while the mass of carrier was 100 mg, 150 mg or 250 mg. That is, the weight ratio (carrier to drug) ranged from 1:1 to 2:1 in the drug loading process. The suspension was ultra-sonicated for more than 45 min. Then, the suspension was placed in a sealed penicillin bottle for 12 h with continuous stirring. Finally, the solvent of the mixture was allowed to dry with stirring. The dried CAR-loaded samples were named MSN/CAR–1 (where the mass ratio of carrier to drug was 1:1), MSN/CAR–1.5, and MSN/CAR–2. When MCN–COOH was applied as the drug carrier, the CAR loading process was the same as that for MSN, and the CAR–loaded samples were named MCN–COOH/CAR–1, MCN–COOH/CAR–1.5, and MCN–COOH/CAR–2. By contrast, we also prepared a physical mixture of MSN (or MCN) and CAR with a mass ratio of 2:1. The physical mixtures were referred to as PM–MSN and PM–MCN–COOH. The drug loading samples were finally passed through a 60-mesh sieve, and kept in a dryer.

#### 3.4.3. Evaluation of Loading Efficiency

The LE values of CAR–loaded samples were measured by a ultraviolet–visible (UV–vis) spectrophotometry. About 20 mg of the CAR–loaded sample was dissolved in 100 mL methanol. The concentration of CAR in the solution was monitored by UV–vis spectrophotometry (UV–2 100, Unico) at 240 nm. The experiments were carried out in triplicate. All experimental data are reported as the mean ± standard deviation.

### 3.5. Dissolution Testing

In vitro dissolution of CAR was performed in pH 1.2 SGF and pH 6.8 SIF. Dissolution testing was conducted using the USP II paddle method with a speed of 100 rpm (900 mL dissolution medium at 37 °C) on an RCZ–8B dissolution tester (Shanghai Huanghai Test Instrument Co., Ltd., China). A sample equivalent to 10 mg CAR was poured into the medium; then, 5 mL of the dissolution medium was taken out for analysis at scheduled time intervals and replaced with 5 mL of fresh fluid. The liquid was filtrated using a 0.80 μm membrane, and the absorbance was measured at 240 nm using a UV–vis spectrophotometer. 

### 3.6. Characterization

The morphology and mesoporous structure of the MSN and MCN nanoparticles were observed by transmission electron microscopy (TEM) images (EM–208S, CSIS, USA). The surface area and pore size distribution of the nanoparticles were characterized using nitrogen adsorption analysis analyzer (V–Sorb 2800P, Gold APP Instrument Corporation, China). The particle sizes and zeta potentials were measured on a Nanosizer (Nano–zs90, Malvern Instruments Ltd., Malvin City, UK). Fourier transform–infrared (FT–IR) spectra were measured on a FT–IR spectrometer (Nicolet 380, Thermo Scientific Ltd., Shanghai, China) using the KBr pellet technique. X–ray diffraction (XRD) was performed on a Siemens D5005 X–ray diffractometer (Karlsruhe, Germany) with Cu–Kα radiation (λ = 1.5418 Å). The differential scanning calorimetry (DSC) experiments were performed by using a calorimeter, (Q2000, TA Instruments, New Castle, NE, USA). Sample was weighed and heated with 10 °C/min under a nitrogen flow of 30 mL/min. 

### 3.7. Cellular Toxicity

Caco-2 cells and NIH-3T3 cells were chosen to evaluate the cytotoxicity of MSN and MCN–COOH carriers. Caco-2 cells were incubated in DMEM with 10% FBS, 1% antibiotics and 1% non-essential amino–acid under a 5% CO_2_ atmosphere at 37 °C. NIH-3T3 cells were incubated in DMEM with 10% FBS, 1% antibiotics under a 5% CO_2_ atmosphere at 37 °C. The in vitro cytotoxicity of MSN and MCN–COOH carriers towards Caco-2 cells and NIH-3T3 cells was measured by MTT assay [32,33]. Generally, caco-2 cells or NIH-3T3 cells were seeded in 96–well plates with a density of 2 × 10^4^ cells/well and incubated for 1 day. The old cell medium was replaced by an equal volume of serum-free medium containing serial concentrations (50, 250, 500, 1000 μg/mL) of nanoparticles. After 1 day of incubation, 50 µL of MTT solution with a concentration of 2 mg mL^−1^ was added to the cell medium and further incubated for 4 h to quantify the living cells. Subsequently, the MTT solution was taken away, and 150 μL of DMSO was added to dissolve the crystalline formazan. Finally, the absorbance was read on a microplate reader (Tecan, Männedorf, Switzerland) at 570 nm.

### 3.8. Gastrointestinal Mucosa Irritation Test

Nine male Sprague–Dawley rats (200 ± 20 g) were randomly divided into three groups. Rats were given MSN or MCN–COOH nanoparticle suspension at a dose of 100 mg/kg every day. Meanwhile, the control group was given the same volume of saline. After a week of this administration regimen, all the animals were sacrificed. Then, their tissues were collected and studied by histological examination.

## 4. Conclusions

The feasibility of two kinds of mesoporous carriers, MSN and MCN, used to increase the dissolution of the insoluble drug CAR, was studied. In addition, the drug-loading and dissolution behaviors of MSN and MCN for CAR were evaluated systematically. MCN–COOH had a higher LE for CAR than MSN for both the two loading methods owing to their strong adsorption capacity and π–π stacking force with CAR. XRD and DSC experiments proved that the optimal mass ratio of MCN–COOH to CAR was 1.5:1, while the optimal mass ratio of MSN to CAR was 2:1, and the drug was in a non-crystalline state in both of the mesoporous carriers. Drug-dissolution testing demonstrated that both MSN and MCN–COOH could markedly improve the dissolution rate of CAR. MCN–COOH displayed a slightly slower dissolution profile when compared with MSN due to the strong interaction between MCN–COOH and CAR. Furthermore, the cell cytotoxicity and gastrointestinal mucosa irritation testing indicated the good biocompatibility of MSN and MCN. We hope that the current work will provide a reference for subsequent research on inorganic materials as oral delivery carriers.

## Figures and Tables

**Table 1:** The N_2_ adsorption/desorption parameters of MSN and MCN–COOH nanoparticles.

Sample	S_BET_ (m^2^/g)	V_P_^a^ (cm^3^/g)	P_d_ (nm)
MSN	1042	0.854	2.6
MCN–COOH	923	0.857	2.6

Note ^a^ V_P_ was calculated by the N_2_ desorption curve with the pore size less than 50 nm.

**Table 2:** The loading efficiencies of CAR loaded samples using solvent evaporation method.

Sample	Mass of Carrier (mg)	Mass of CAR (mg)	Sample Name after CAR Loading	Drug Loading (%)	Theoretical LE (%)
MSN	100	100	MSN/CAR–1	39.5±3.0	50.0
	150	100	MSN/CAR–1.5	34.0±2.8	40.0
	200	100	MSN/CAR–2	28.2±2.3	33.3
MCN–COOH	100	100	MCN–COOH/CAR–1	42.9±2.7	50.0
	150	100	MCN–COOH/CAR–1.5	35.3±3.0	40.0
	200	100	MCN–COOH/CAR–2	27.2±0.9	33.3
